# Semantic Edge Based Disparity Estimation Using Adaptive Dynamic Programming for Binocular Sensors

**DOI:** 10.3390/s18041074

**Published:** 2018-04-03

**Authors:** Dongchen Zhu, Jiamao Li, Xianshun Wang, Jingquan Peng, Wenjun Shi, Xiaolin Zhang

**Affiliations:** 1 Bio-Vision System Laboratory, State Key Laboratory of Transducer Technology, Shanghai Institute of Microsystem and Information Technology, Chinese Academy of Sciences, Shanghai 200050, China; jmli@mail.sim.ac.cn (J.L.); xswang@mail.sim.ac.cn (X.W.); pengjq@mail.sim.ac.cn (J.P.); wjs@mail.sim.ac.cn (W.S.); xlzhang@mail.sim.ac.cn (X.Z.); 2 University of Chinese Academy of Sciences, Beijing 100049, China; 3 School of Information Science and Technology, ShanghaiTech University, Shanghai 201210, China

**Keywords:** sparse stereo matching, disparity estimation, semantic edge, dynamic programming, binocular sensor

## Abstract

Disparity calculation is crucial for binocular sensor ranging. The disparity estimation based on edges is an important branch in the research of sparse stereo matching and plays an important role in visual navigation. In this paper, we propose a robust sparse stereo matching method based on the semantic edges. Some simple matching costs are used first, and then a novel adaptive dynamic programming algorithm is proposed to obtain optimal solutions. This algorithm makes use of the disparity or semantic consistency constraint between the stereo images to adaptively search parameters, which can improve the robustness of our method. The proposed method is compared quantitatively and qualitatively with the traditional dynamic programming method, some dense stereo matching methods, and the advanced edge-based method respectively. Experiments show that our method can provide superior performance on the above comparison.

## 1. Introduction

Depth acquisition is crucial for environment perception of intelligent devices, and it plays an important role in various applications, such as 3D reconstruction, Augmented Reality (AR), and vehicle navigation. Sensors that can be used to obtain depth include laser sensors, visual sensors, and radar sensors. Nowadays, laser and radar sensors have been widely used. With the progress of computer vision algorithms, visual sensors represented by binocular cameras show strong competitiveness, which are becoming an important research direction of automatic driving. Binocular sensors acquire depth by estimating the disparities, which aims to calculate the coordinate difference between corresponding points in the rectified stereo images. According to the principle of triangulation, the depth of each point is easy to measure with its disparity, since the disparity is inversely proportional to the distance from cameras.

Numerous algorithms have been widely studied to solve this problem over the past decades. According to the density of the generated disparity maps, these methods can be divided into two categories: dense stereo matching and sparse stereo matching. The dense matching methods strive to obtain each pixel’s disparity. Although the traditional methods, such as Graph Cuts (GC) [1,2], Semi-Global Matching (SGM) [3], and the emerging approaches based on deep learning, like MC-CNN [4,5], have achieved high accuracy, there is an intractable problem of heavy computation in these dense methods. While the sparse methods based on points and lines are less studied than dense matching in recent years, they are not given up by the researchers who seek the balance between accuracy and efficiency because of the advantage of less computational complexity.

Point-based sparse methods have been effectively applied for the richly textured regions, but they fail in textureless areas [6]. The methods based on straight and curved lines possess universality, but most of the lines or edges extraction methods are based on low-level features with the lack of scene understanding. Calculating only these pixels’ disparities cannot guarantee that the basic demands for visual navigation are satisfied, which means that these algorithms have difficulties with being applied in practice.

In this paper, we propose a new sparse disparity estimation method for outdoor navigation. The proposed approach focuses on the pixel matching of semantic edges because those contours not only segment out objects in the scene, but also give them different meanings. Estimating disparities of these semantic edges can meet the basic requirements of recognition, location, and obstacle avoidance in navigation completely. We also put forward an adaptive dynamic programming algorithm, which can automatically select the parameters to improve the robustness of our method.

We organize the remainder of this paper as follows. Section 2 introduces the related work about the stereo matching. The specific matching approach will be introduced in detail in Section 3. Some qualitative and quantitative experiments are demonstrated in Section 4. Section 5 makes a brief summary at the end of this paper.

## 2. Related Work

According to [7], the dense stereo matching can be generally divided into four steps: cost calculation, cost aggregation, disparity optimization, and optionally refinement. In fact, plenty of sparse methods also follow these four steps.

Absolute Difference (AD), the Sum of the Absolute Difference (SAD), Census transform (Census), and Normalization Cross Correlation (NCC) are all classic cost calculation methods [8]. Then [9] found that the combination of different costs can draw on each other’s strength. They combined AD and Census to get a better result. This idea has been widely applied and we will also draw lessons from it. With the development of deep learning, the methods of calculating cost using neural networks, like [5], have also achieved excellent results in dataset tests. However, these methods are still limited in the ground truth of datasets for training. Our method still will choose the classic costs.

There are a variety of ways to aggregate costs. Mean filtering, median filtering, etc. can be regarded as aggregation with a window of fixed size. The more popular one is the adaptive aggregation method with irregular windows. Ke Zhang et al. [10] proposed the cross-based aggregation that determines the support window by comparing the similarity between the surrounding pixels and the anchor pixels, and suggested considering the left and right images together. However, this method is time-consuming. In this paper, we do not take the step of cost aggregation, but choose to increase the window size appropriately.

The disparity optimization has been widely studied, creating a variety of algorithms. Winner-Take-All (WTA) is the simplest one without considering global information. Dynamic programming (DP) has been widely used in dense and sparse matching, and is the method to be adopted in this paper. Belief propagation (BP) and Graph Cuts (GC) based on Markov random field also have excellent performance. In recent years, the most widely used is Semi-Global Matching (SGM). This algorithm employs multi-path one-dimensional optimizations to solve two-dimensional optimization problem by designing the constraints between each anchor pixel and its neighborhoods. This method is still very competitive since being proposed, and some results based on SGM will be compared with our method in Section 4.

In this paper, we do not modify the disparities obtained by optimization, so we will not introduce the refinement methods. Next, we will mainly discuss the related edge-based methods. They can also be divided into two kinds: based on straight lines and based on curved edges. Line-based approaches assume that lots of straight lines can be detected in the images. They have achieved results in stereo [11] and Structure From Motion (SFM) [12]. However, when there are fewer lines detectable in the scene, such as our outdoor navigation scenarios, these approaches are useless.

Curve-based approaches are improved with the development of edge extraction methods. In [13], the positions of edges are detected by differentiating and then linked to connected edges. Then, dynamic programming is used in intra- and inter-scanline search with edge-delimited intervals as elements to be matched. The advantage of [13] is that the inter-scanline constraint is taken into consideration, but the constraint is so strong that only the edges crossing multiple scanlines are preserved, without considering the horizontal edges. Moreover, the inter-scanline search results in a high cost of computation.

In [14], a sparse BP algorithm is designed to match the nodes of the mesh of left image, formed by edge and non-edge pixels, with the all pixels of the right image. Compared with the dense BP, the computational complexity of [14] is reduced. However, only the number of pixels to be matched is reduced, the matching search range of each pixel is not changed. The possible mismatches of dense BP algorithm cannot be avoided yet.

The methods proposed in [15,16,17] are all based on canny edges. In [15], some corresponding corner points are selected as seed points, and then the correspondences between edges around those seed points are determined. Seed points are helpful to narrow the range of edges to be matched, but the mismatches of seeds can lead to edge matching errors.

The methods of cost calculation and aggregation are the same in [16,17]. They choose the minimum of both asymmetric pixel-strips on either side of a candidate edge as the matching cost of the edge. In [16], a confidence-based refinement algorithm is applied after the initial WTA process, while a dynamic programming algorithm is implemented on each edge segment in [17]. They both take into consideration the consistency and smoothness of edges. However, the description of each edge pixel can be ambiguous, which is easy for causing a mismatch.

Subhayan Mukherjee et al. [18] uses K-means clustering to segment the left image, and refines the segment boundaries by morphological filtering. Then, SAD and WTA are used to match the edges of the left image with the full right image. Finally, a dense disparity map can be obtained by disparity propagation. The idea of using K-means segment boundaries is novel, but the value of k needs to be specified according to different images. In addition, the defects of WTA and sparse-dense matching mentioned above do not get addressed.

Dexmont Peña et al. [19] extended the Edge Drawing (ED) algorithm on stereo pairs, matched only a few anchor points and then propagated disparities along the edges. Using the proposed method, the number of computations can be greatly decreased. However, this method is highly dependent on the matching accuracy of anchor points. A tiny match error can be propagated to the entire edge.

With the development of deep learning, the semantic edge extraction method has been researched. Gedas Bertasius et al. [20] used VGG [21] to detect edges and then used semantic segmentation networks to classify edges. The newest [22] proposed an end-to-end network that detected semantic edge with multi-category labels. Our method will be based on Ref. [22]’s edges to estimate disparities, considering edge points as candidates to be matched and using an improved dynamic programming algorithm to obtain the global optimal solution for each scanline, which will be described in detail below.

## 3. Proposed Method

**Figure 1:**
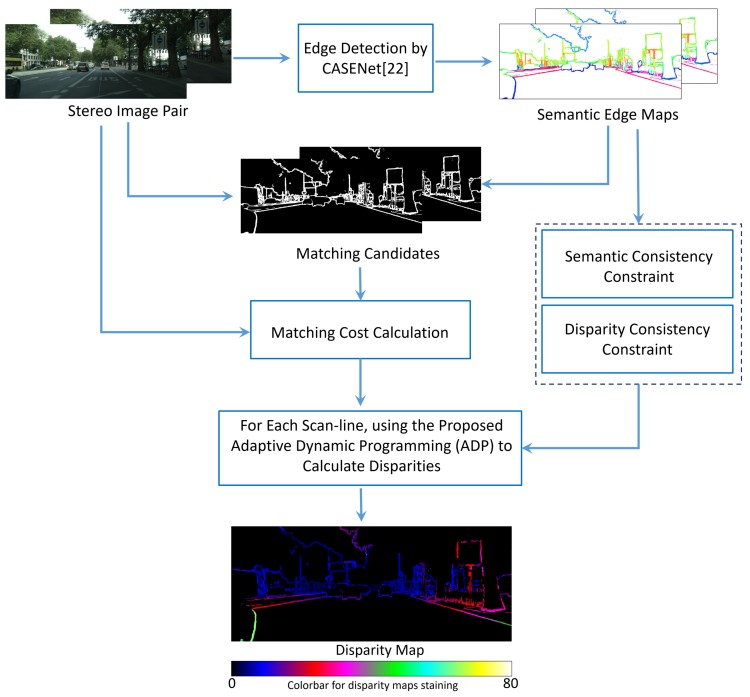
Pipeline of our proposed method.

In this section, our proposed Semantic Edge Matching (SEM) method is described in detail, which is illustrated in Figure 1. The input of our method is a calibrated and rectified stereo image pair, and the output is a disparity map, which is calculated sparsely for semantic edges in the scene. For this purpose, we first use the CASENet to detect the semantic edges of the left and right images, respectively. These extracted edge pixels are candidates to be matched in this paper. In order to find the correspondence between pixels in stereo images, we attribute the matching of each scan-line’s pixels to the alignment of two sequences, and then this problem can be solved by a dynamic programming paradigm. Before solving the matching problem, we first need to measure the similarity between the matching candidates, and this is the calculation of the cost. In Section 3.2, we will introduce the cost computing methods used in experiments. Next, we propose an Adaptive Dynamic Programming (ADP) algorithm because the traditional dynamic programming method has the difficulty of selecting empirical parameters, which should not be the same for different scenes. ADP makes use of the potential consistency in stereo image pairs to select optimal parameters adaptively, and two kinds of consistencies can be applied in it. The results of our method will be analyzed qualitatively and quantitatively in Section 4. In the following subsections, we will elaborate on each step of our method.

### 3.1. Semantic Edge Detection and Semantic Edge Matching Formulation

As a kind of important feature of images, edges are widely used in a series of computer vision problems. There are many existing methods of edge extraction, from the low-level method using simple convolutional filters such as Sobel [23] and Canny [24] to the high-level method with semantic neural networks such as High-for-Low approach (HFL) [20]. In this paper, our selected method is CASENet [22], which is a semantic edge detection network with category-aware. There are two main reasons why we choose this method:

1. CASENnet differs from the traditional methods in that it detects not the low-level edges, but the external contours of each object in the image. Since the lines inside of the object are not detected, there are fewer matching candidates in this paper, which can reduce the interference of some similar edges.
2. Unlike the traditional edge detection to solve a binary classification problem, in which a pixel belongs to edges or not, CASENet employs a new modeling approach that allows pixels in contours belonging to more than one category. In other words, CASENet detects object contours with semantic meaning. Therefore, only matching these pixels can satisfy the basic ranging requirement in navigation because the distance of each target in the scene can be calculated. At the same time, the multi-contour features of the edge pixels also make it possible for each pixel to measure the similarity in semantic space. This feature will be taken advantage of in Section 3.3.

An example of CASENnet’s result is shown in Figure 3, and the meanings of different colors are the same as in [22]. There is still one thing with which we should be concerned. The width of each edge extracted by CASENet is several pixels rather than one pixel. With these edge pixels as the matching candidates, we formulate the semantic edge matching problem in this paper as:

Given two arrays $\left\{L_{1},L_{2},...,L_{m}\right\}$ and $\left\{R_{1},R_{2},...,R_{n}\right\}$ for each pair of scan-lines in left image $I_{L}$ and right image $I_{R}$, we aim to find the alignment M of minimum cost C(M), in which:

(1) $L_{i}=\left(x_{L_{i}},y_{L_{i}}\right)$ and $R_{j}=\left(x_{R_{j}},y_{R_{j}}\right)$ are the coordinates of pixels to be matched in left and right images respectively, and there exists $y_{L_{i}}=y_{R_{j}}$.
(2) M is a set of pairs $\left\{L_{i},R_{j}\right\}$ in order. More specifically, each item in left array $\left\{L_{1},L_{2},...,L_{m}\right\}$ has at most one corresponding item in right array $\left\{R_{1},R_{2},...,R_{n}\right\}$ and vice versa, and if there are two pairs $\left\{L_{i_{1}},R_{j_{1}}\right\}$ and $\left\{L_{i_{2}},R_{j_{2}}\right\}$, there must be $j_{1}>j_{2}$ when $i_{1}>i_{2}$.
(3) The cost of M is defined as:

(1) $$C\left(M\right)=\sum_{\{L_{i},R_{j}\}\in M}C_{L_{i},R_{j}}+\sum_{L_{i}orR_{j}\mathrm{unmatched}}C_{\mathrm{gap}},$$

where $C_{L_{i},R_{j}}$ is the color cost between $L_{i}$ and $R_{j}$, which represents the similarity between the two pixels; $C_{\mathrm{gap}}$ is the unmatched cost if $L_{i}$ or $R_{j}$ has no matched item.

The formulated problem can be solved by dynamic programming paradigm. However, the traditional dynamic programming method suffers parameters selection difficulty. Therefore, we propose an improved method with an adaptive parameter to solve the above problem. Then, the disparity *d* of each pixel in left image can be calculated by

(2) $$d_{L}=x_{L_{i}}-x_{R_{j}}.$$

Different from other stereo matching methods, we do not set the value of maximum disparity in our method. However, in order to show the results more clearly, we set a maximum disparity for consistent staining. The color range is shown as Figure 1.

### 3.2. Color Cost Calculation

The first and most important step of stereo matching is to describe the potential property of each waiting-for-matched pixel effectively, and then scale the comparability between different pixels. That is to say, the matching cost is computed at first to represent the similarity of pixels. We will introduce three kinds of matching costs below, which will be used in our method.

The first one is SAD, which can be implemented quickly by using the mean filtering on AD. They both assume that the brightnesses of corresponding pixels are consistent. The mathematical expression of AD and SAD is as Equations (3) and (4), respectively:

(3) $$C_{\mathrm{AD}}\left(x,y,d\right)=\left|I_{L}\left(x,y\right)-I_{R}\left(x-d,y\right)\right|,$$

(4) $$C_{\mathrm{SAD}}\left(x,y,d\right)=\sum_{\left(x^{\prime },y^{\prime }\right)\in N_{\left(x,y\right)}}\left|I_{L}\left(x^{\prime },y^{\prime }\right)-I_{R}\left(x^{\prime }-d,y^{\prime }\right)\right|,$$

where $N_{\left(x,y\right)}$ is the neighborhood of pixel (x,y), and it is usually a rectangular window with size $W_{SAD}\times H_{SAD}$. The $W_{SAD}$ is the width of $N_{\left(x,y\right)}$ and the $H_{SAD}$ is the height.

**Figure 2:**
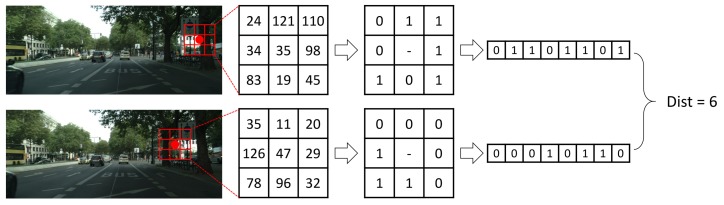
Example of Census cost. The similarity is measured by Hamming distance.

Another cost is Census, which encodes each pixel into a binary descriptor and calculates the similarity between different pixels by Hamming distance. It encodes the brightness relation between the anchor pixel and its neighbors rather than the brightnesses themselves, so it is robust to the radiometric change. However, it has ambiguities in repetitive and similar structures. The mathematical expressions of it is as Equation (5):

(5) $$C_{\mathrm{Census}}\left(x,y,d\right)=\mathrm{HammingDistance}\left(\rho \left(I_{L}\left(x,y\right)\right),\rho \left(I_{R}\left(x-d,y\right)\right)\right),$$

in which, $\rho \left(I\left(x,y\right)\right)={⊗}_{\left(x^{\prime },y^{\prime }\right)\in N_{\left(x,y\right)}}\left\{\begin{array}{cc} 1 & I\left(x^{\prime },y^{\prime }\right)>I\left(x,y\right)\\ 0 & otherwise \end{array}\right.$, ⊗ denotes concatenation and $N_{\left(x,y\right)}$ is also the neighborhood of pixel (x,y) with a rectangular window size $W_{Census}\times H_{Census}$. An example of $C_{\mathrm{Census}}$ is shown as Figure 2.

The last cost is the combination of $C_{\mathrm{SAD}}\left(x,y,d\right)$ and $C_{\mathrm{Census}}\left(x,y,d\right)$ shown as Equation (6), which is similar to the AD-Census Cost in [9]. However, we reduced the number of parameters to be determined, which improves the robustness of our method. Only one constant coefficient α should be given:

(6) $$C_{\mathrm{SADCen}}\left(x,y,d\right)=C_{\mathrm{SAD}}\left(x,y,d\right)+\alpha \times C_{\mathrm{Census}}\left(x,y,d\right).$$

In fact, the SAD cost and Census cost we used in this paper are normalized. With the above costs, the $\left\{C_{L_{i},R_{j}}\right\}$ in Equation (1) are prepared. The results of our method using different kinds of costs will be compared in Section 4.

### 3.3. Adaptive Dynamic Programming

**Figure 3:**
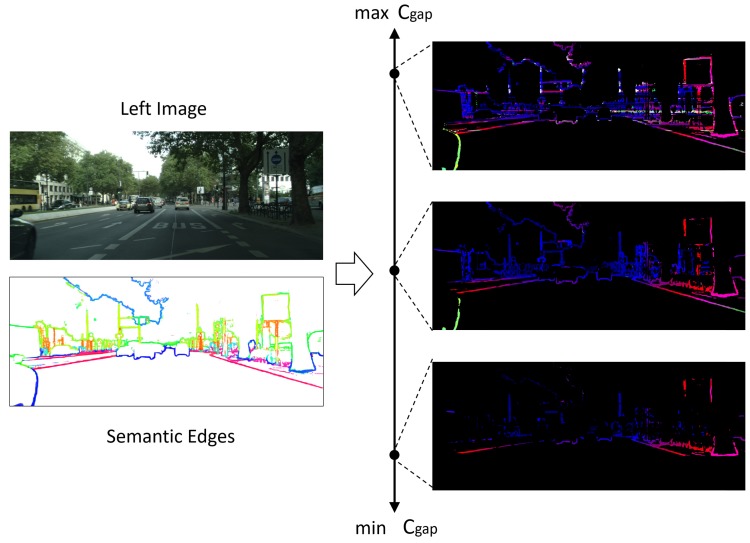
Influence of different $C_{\mathrm{gap}}$s. Too small $C_{\mathrm{gap}}$ will cause lots of unmatched pixels, while too large $C_{\mathrm{gap}}$ will cause lots of mismatches.

The minimization problem of Equation (1) can be solved by dynamic programming paradigm:

(7) $$OPT\left(i,j\right)=\left\{\begin{array}{cc} j\times C_{\mathrm{gap}} & \mathrm{if}\;i=0,\\ \min\left\{\begin{array}{c} C_{L_{i},R_{j}}+OPT\left(i-1,j-1\right)\\ C_{\mathrm{gap}}+OPT\left(i-1,j\right)\\ C_{\mathrm{gap}}+OPT\left(i,j-1\right) \end{array}\right. & \mathrm{otherwise},\\ i\times C_{\mathrm{gap}} & \mathrm{if}\;j=0, \end{array}\right.$$

where OPT(i,j) is the minimum cost of aligning arrays $\left\{L_{1},L_{2},...,L_{i}\right\}$ and $\left\{R_{1},R_{2},...,R_{j}\right\}$. The $C_{L_{i},R_{j}}$ is calculated as Section 3.2. The $C_{\mathrm{gap}}$ is usually an empirical constant, which plays an important role but is hard to choose. When $C_{\mathrm{gap}}$ is too large, it will cause more mismatches; and when $C_{\mathrm{gap}}$ is too small, it will cause most pixels unmatched. A moderate value is very difficult to select in applications, as shown in Figure 3.

In order to improve the robustness, we propose an improved dynamic programming algorithm, which makes use of the underlying consistency between stereo images to select the value of $C_{\mathrm{gap}}$ adaptively. We put forward two kinds of consistency constraints that can be used: disparity consistency constraint and semantic consistency constraint. We also try to utilize them at the same time. However, the experiments in Section 4 show that it is not necessary because the semantic consistency constraint in this case is much stronger than the disparity constraint.

#### 3.3.1. Adaptive Dynamic Programming with Disparity Consistency Constraint

Once the correspondence between two pixels in stereo images is determined, Equation (8) must exist because of the consistency between left and right disparity maps. In stereo matching, it is called LRC (Left-Right Consistency) check and is usually used for mismatch detection:

(8) $$d_{L}\left(x,y\right)=d_{R}\left(x-d_{L}\left(x,y\right),y\right).$$

Actually, this method can not detect all mismatches. Although the probability is low, there still exist consistent errors in left and right disparities. However, it is still the most popular detection method since it is easy to implement and is effective in practice. In this part, we count the number of pixel pairs that satisfy Equation (8) to obtain the value of $C_{\mathrm{gap}}$ dynamically, The details are shown in Algorithm 1.

**Figure 4:**
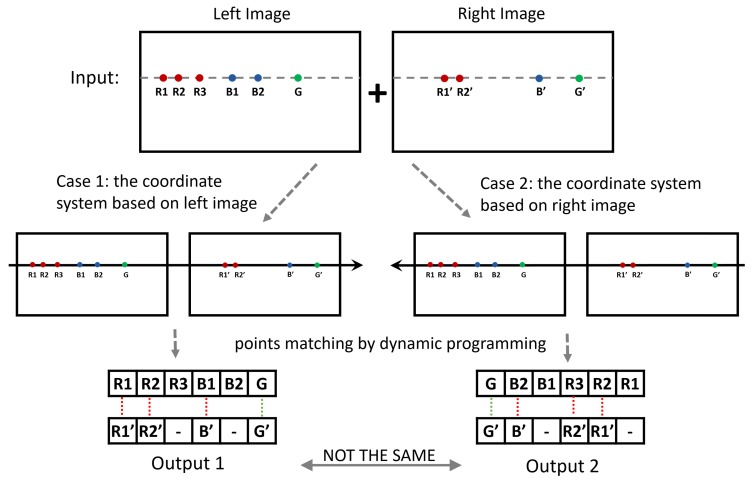
Example of different matching orders. For the same sequences to be matched, the results will be different if the coordinate systems are not the same. The red dotted lines in the results indicate inconsistent pairs on both sides.

The *MatchingCost* function aims to calculate each similarity between $L_{i},i\in \left\{1,2,...,m\right\}$ and $R_{j},j\in \left\{1,2,...,n\right\}$, so the numbers of items in $\left\{C_{L_{i},R_{j}}\right\}$ and $\left\{C_{R_{j},L_{i}}\right\}$ are m×n, respectively. In effect, $\left\{C_{R_{j},L_{i}}\right\}$ can be obtained from $\left\{C_{L_{i},R_{j}}\right\}$ without recalculating. Generating the disparity map of left image is to try to find a matching relationship between $\left\{L_{1},L_{2},...,L_{m}\right\}$ and $\left\{R_{1},R_{2},...,R_{n}\right\}$, while generating the disparity map of the right image is to try to find a matching relationship between $\left\{R_{n},R_{n-1},...,R_{1}\right\}$ and $\left\{L_{m},L_{m-1},...,L_{1}\right\}$. With each image as the base, the coordinate system and the matching order are different. In the case of dynamic programming, different matching orders may result in different results. An example is given in Figure 4.

Instead of fixing the value of $C_{\mathrm{gap}}$, we provide a range of values for the search of each scan-line’s optimal $C_{\mathrm{gap}}$. When the Census cost is chosen, the bottom of the search range ($\lambda_{min}$) will be set as 0, the top of the search range ($\lambda_{max}$) will be set as $W_{Census}\times H_{Census}$, and the search interval ($\lambda_{inter}$) will be set as 1. This is due to the binary descriptor of Census. The maximum, the minimum, and the smallest interval of Census cost are 0, the length of descriptor, and 1, respectively. When the SAD cost is selected, the search interval ($\lambda_{inter}$) will be set as 0.01, and if $\left\{C_{L_{i},R_{j}}\right\}$ is sorted in ascending order, the $\lambda_{min}$ and $\lambda_{max}$ will be set as $C_{\theta_{min}}$ and $C_{\theta_{max}}$ picked from the ordered cost $\left\{C_{1},C_{2},...,C_{m\times n}\right\}$. The $\theta_{min}$ and $\theta_{max}$ is computed as Equations (9) and (10) based on empirical conjecture and experimental verification rather than set as the theoretical minimum and maximum, since the distribution of SAD cost is not as predictable as Census cost. Ideally, there should be at most $l_{min}=min\left\{m,n\right\}$ values in the cost that are distinct from the other values because there are at most $l_{min}$ pairs of matches. However, in practice, the true match may occur at the second or third smallest cost. Therefore, we set $\lambda_{min}$ as the $k_{1}l_{min}$-th cost in $\left\{C_{1},C_{2},...,C_{m\times n}\right\}$ and the extent size ($\lambda_{max}-\lambda_{min}$) as $k_{2}l_{min}$. $k_{1}\in \left\{1,2,3\right\}$ and $k_{2}\in \left\{1,2,3\right\}$ in this paper:

(9) $$\begin{array}{cc} \max\theta_{min}\;s.t.\; & \theta_{min}\in \left\{3\times l_{min},2\times l_{min},l_{min}\right\}\\ & \theta_{min}<m\times n,l_{min}=min\left\{m,n\right\}, \end{array}$$

(10) $$\begin{array}{cc} \max\theta_{max}\;s.t.\; & \theta_{max}-\theta_{min}\in \left\{3\times l_{min},2\times l_{min},l_{min}\right\}\\ & \theta_{max}<m\times n,l_{min}=min\left\{m,n\right\}. \end{array}$$

The *DPMatching* function computes the optimal value of Equation (7) and finds the solution. Since the dynamic programming algorithm is very classic, we will not give details in this paper. The *DisparityComputing* function converts the alignment $M_{LR}$ to disparities $\left\{d_{L}^{1},d_{L}^{2},...,d_{L}^{p}\right\}$ with Equation (2), and the disparities $\left\{d_{R}^{1},d_{R}^{2},...,d_{R}^{q}\right\}$ can be obtained in a similar way. The *LRC* function eliminates those matches that do not satisfy Equation (8). The disparities $\left\{d_{L}^{1},d_{L}^{2},...,d_{L}^{k}\right\}$ that achieve the maximum consistency between left and right arrays are obtained by our algorithm, and the $C_{\mathrm{gap}}$ at this time is the optimal value. Hence, one can see that the $C_{\mathrm{gap}}$ is self-adapting for each scan-line in different images.

**Algorithm 1:** Adaptive Dynamic Programming with Disparity Consistency Constraint for Each Pair of Scan-Lines in Stereo Images.
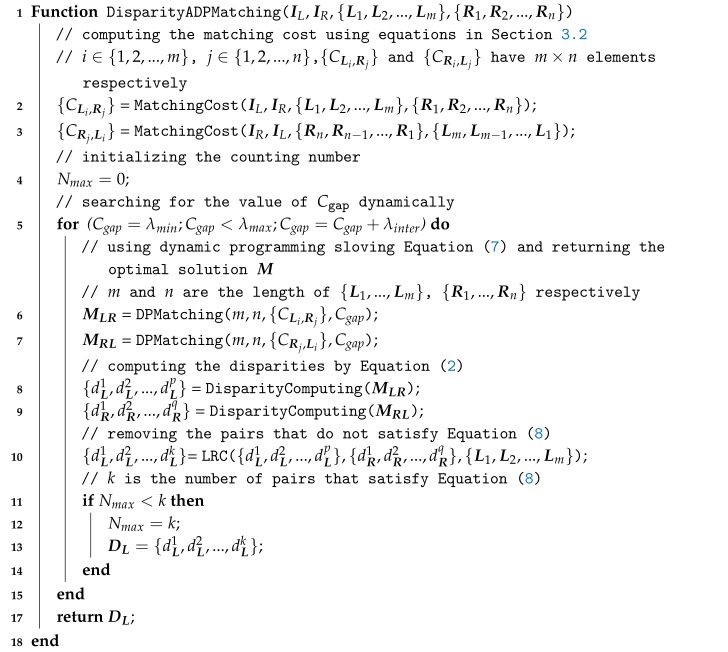


#### 3.3.2. Adaptive Dynamic Programming with Semantic Consistency Constraint

In addition to the disparity consistency mentioned above, there should also exist semantic consistency between the left and right images, shown as Equation (11), since we use the semantic edge pixels as matching candidates. We name this algorithm as Semantic Left-Right Consistency (SLRC) check. Assuming the output of CASENet is *E*, $E\left(x,y\right)={\left\{0,1\right\}}^{s}$, 1 means belonging to a class, and 0 has the opposite meaning. The similarity between different vectors can be measured by Hamming Distance. If only the equality is determined, the vectors can also be converted to decimal digits to compare:

(11) $$E_{L}\left(x,y\right)=E_{R}\left(x-d_{L}\left(x,y\right),y\right).$$

By replacing the disparity consistency constraint with the semantic consistency constraint, Algorithm 2 can be obtained. The functions, which include *MatchingCost*, *DPMatching*, *DisparityComputing*, and the parameters, which include $\lambda_{min}$, $\lambda_{max}$, $\lambda_{inter}$ are all the same as the ones in Algorithm 1. Similar to the *LRC* function, the *SLRC* function eliminates those matches that do not satisfy Equation (11). The greatest difference between Algorithms 1 and 2 lies on the right disparity map. Algorithm 2 just needs the left disparity map to determine the semantics of matching pixels. It makes Algorithm 2 faster than Algorithm 1.

Additionally, it is natural to merge the two algorithms using disparity and semantic constraints at the same time, and we did it in Section 4. However, the results are the same as using semantic constraints only in our experiments.

**Algorithm 2:** Adaptive Dynamic Programming with Semantic Consistency Constraint for Each Pair of Scan-Lines in Stereo Images.
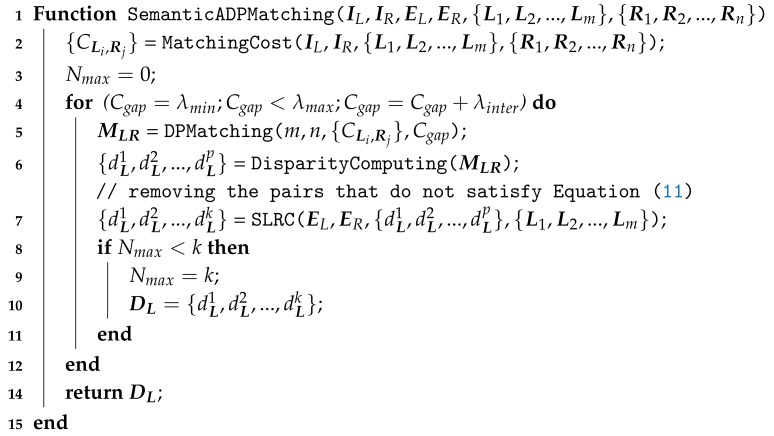


## 4. Experiments

In this section, we conduct experiments to study the performance of our method. We will introduce the dataset we used and the evaluation indicators. Then, we will discuss the performance of different window sizes, different costs, and our proposed method separately. We implemented our algorithms on the MATLAB platform with a laptop of Intel Core-i7 CPU (4 cores @2.20 GHz) and 16 GB RAM. No parallel technology is used to accelerate our algorithms.

### 4.1. Dataset and Evaluation Methods

In existing public datasets, there is no one that has plenty of semantic annotations for outdoors and dense enough ground truth of disparities at the same time. It has caused some difficulties for our quantitative evaluation. The candidate datasets considering by us include KITTI Stereo [25,26] and Cityscapes [27]. Both of them are captured by driving cars around the cities and contain the stereo images. KITTI provides the disparity ground truth by a Velodyne laser scanner and a GPS localization system [25]. However, the ground truth does not cover each whole image. The upper portion of the image has no ground truth, and the ground truth of the lower part is interlaced. The results of our method are sparse for the entire images. There are very small overlaps between our results and ground truth. In addition, KITTI has few semantic annotations provided by other researchers, like [28,29]. Most of these annotations are not in high quality. Cityscapes is a large-scale dataset with high quality annotations of 5000 frames and weakly annotated of 20,000 frames [27]. The output of CASENet on it is more reliable, which is the most important premise of our algorithm implementation. Although this dataset contains no disparity ground truth, we can make the ground truth using reliable feature points matching. As a consequence, we chose Cityscapes to evaluate our experiments. More specifically, we pick out the Berlin data of 544 frames from Cityscapes as our evaluation dataset.

**Figure 5:**
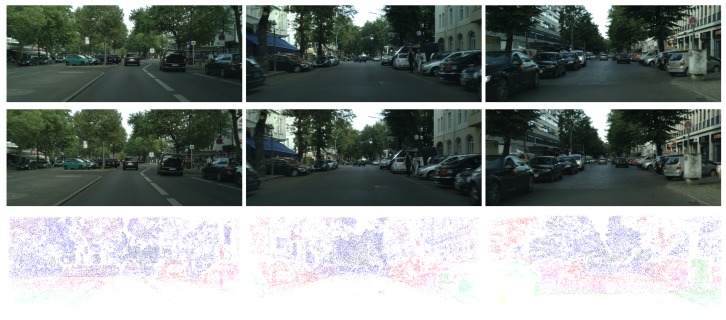
Examples of our ground truth. From top to bottom: the left images, the right images, and our ground truth.

We used a variety of local feature extraction methods for interest points detecting, extracting, and matching. SURF (Speed Up Robust Feature) [30], Harris [31], FAST (Features from Accelerated Segment Test) [32], and BRISK (Binary Robust Invariant Scalable Keypoints) [33] are all taken into account. Then, we applied GMS (Grid-based Motion Statistics) [34] to distinguish inliers from outliers quickly. We can get a set of ground truth, in which there are about 70,000 truth values for each image pairs in our dataset. Some examples are shown in Figure 5. With the ground truth made by us, some quantitative evaluation can be done for reference. We compute the following two measures in the remaining part of this paper:

(12) $$Density=\frac{N_{D}}{N_{I}},$$

where $N_{D}$ is the number of the pixels with values in disparity maps, and $N_{I}$ is the number of each whole image’s pixels. In our experiments, $N_{I}$ is equal to 825×2009:

(13) $$Error=\frac{\sum_{(x,y)\in D\cap \mathrm{GT}}\left(\right|d\left(x,y\right)-d_{\mathrm{GT}}\left(x,y\right)\left|\right)>\delta_{d}}{N_{D\cap \mathrm{GT}}},$$

where $N_{D\cap \mathrm{GT}}$ is the number of the pixels that have values in disparity maps and ground truth maps simultaneously; $\delta_{d}$ is an error tolerance. We set the $\delta_{d}$ as 3, 2, and 1, respectively.

### 4.2. Performance of Different Window Sizes

The costs used in this paper are simple local ones, and there is no cost aggregation to enhance the global information. In order to ensure that each candidate can be characterized well, it is crucial to choose the appropriate window size. If the window is too small, it is not enough to support each pixel; if it is too big, the calculation time will be increased. Therefore, we compare the effects of different window sizes through experiments.

**Figure 6:**
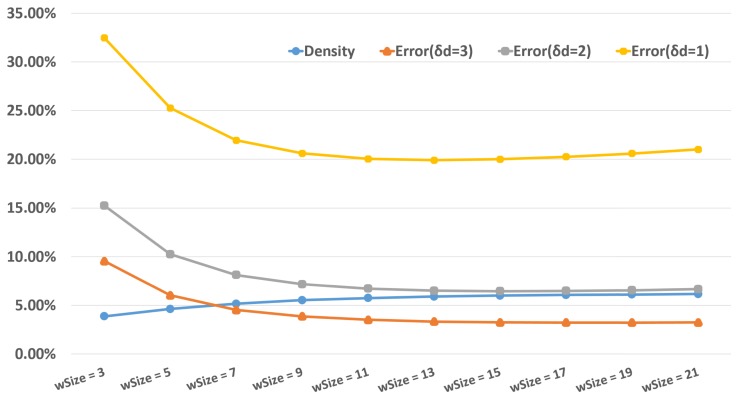
Effects of different window sizes.

We use SAD cost and our proposed ADP algorithm with SLRC in this subsection. The corresponding performance indicators are recorded by changing the window size. To facilitate the experiments, we set the height $H_{SAD}$ and width $W_{SAD}$ to the same value wSize. The results are collected in Figure 6. Before wSize reaches 15, Density is gradually increasing and Errors are decreasing with the increase of wSize. After that, Density is still increasing, but Errors are increasing slightly. Therefore, we set the $H_{SAD}$ and $W_{SAD}$ as 15 in our next experiments. Actually, the $H_{Census}$ and $W_{Census}$ are set as 15 too.

### 4.3. Performance of Adaptive Dynamic Programming

**Figure 7:**
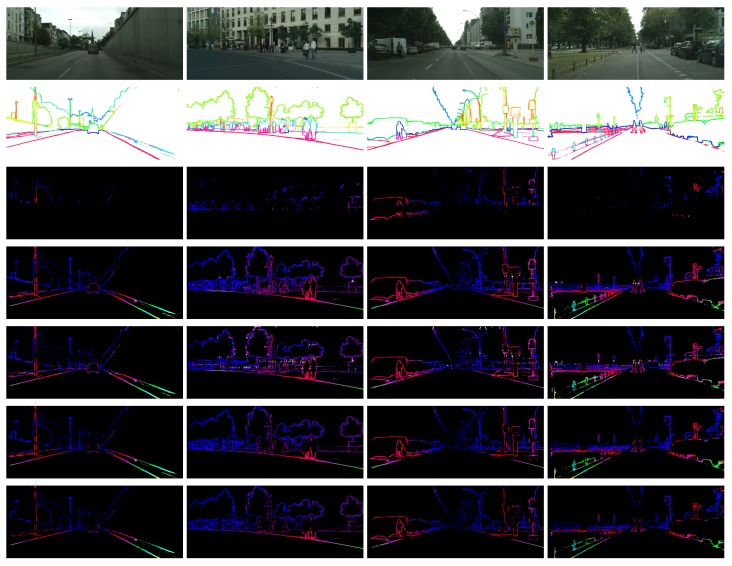
Examples of comparison between our method and the traditional dynamic programming algorithm. From top to bottom: the left images, the semantic edges, the disparity maps of the following algorithms separately: DP($C_{\mathrm{gap}}=0.0005$), DP($C_{\mathrm{gap}}=0.1$), DP($C_{\mathrm{gap}}=0.9$), ADP(LRC), ADP(SLRC).

As previously mentioned, to determine the value of $C_{\mathrm{gap}}$ is a key challenge in the dynamic programming algorithm. $C_{\mathrm{gap}}$s that are too large or too small will result in unsatisfactory results (see in Figure 3 and Figure 7). To prove the validity of our proposed algorithm, we assign several values to $C_{\mathrm{gap}}$ to compare with our adaptive algorithms. The cost we used in this subsection is still SAD.

Table 1 details the comparisons. When $C_{\mathrm{gap}}$ is equal to 0.0005, the Density is very low. It means that 0.0005 is so small that many pixels cannot find correspondences. Although the Errors are low in this case, the results can not be used in practice. When $C_{\mathrm{gap}}$ is equal to 0.1 or 0.9, the Density is high, but the Errors are high too. Our proposed adaptive programming algorithm, no matter which constraint is used, can guarantee a relatively high Density and relatively low Errors. The algorithm with disparity constraint can obtain denser results, while the algorithm with semantic constraint can get higher accuracy. The results of the algorithms with semantic constraint and combined constraint are exactly the same. We will not consider the algorithm with combined constraints in the later experiments. From Table 1, we can also see that the iterative step of our proposed adaptive programming increases the runtime. As we inferred in the previous section, our proposed ADP algorithm with SLRC consumes less time than with LRC.

### 4.4. Performance of Our Proposed Method

In this subsection, we will try the combinations of different costs and adaptive dynamic programming algorithms with different constraints, and then compare our results with other methods.

The six combinations of our method are shown in Table 2 and Figure 9. As you can see, the accuracy of using SAD cost is superior to that of using Census cost when the same ADP algorithm is employed. The combined cost has the best performance by absorbing the advantages of both. In the case of using the same kind of cost, the accuracy of ADP with SLRC is higher than that of ADP with LRC. The advantages of SLRC are especially prominent with Census cost. The average computation time of ADP with Census cost is much higher than others. One reason is the slow calculation of Census cost, and the other one is the wide range of $C_{\mathrm{gap}}$ when Census cost is used.

In addition to comparing with the traditional dynamic programming algorithm, we will make further efforts to compare our method with other ones in order to demonstrate the effectiveness of our method. So far as we know, there is no one only matching the extracted edges of CASENet as we did in this paper, which makes difficulties for our comparison. For this, we have developed two schemes as below.

**Figure 8:**
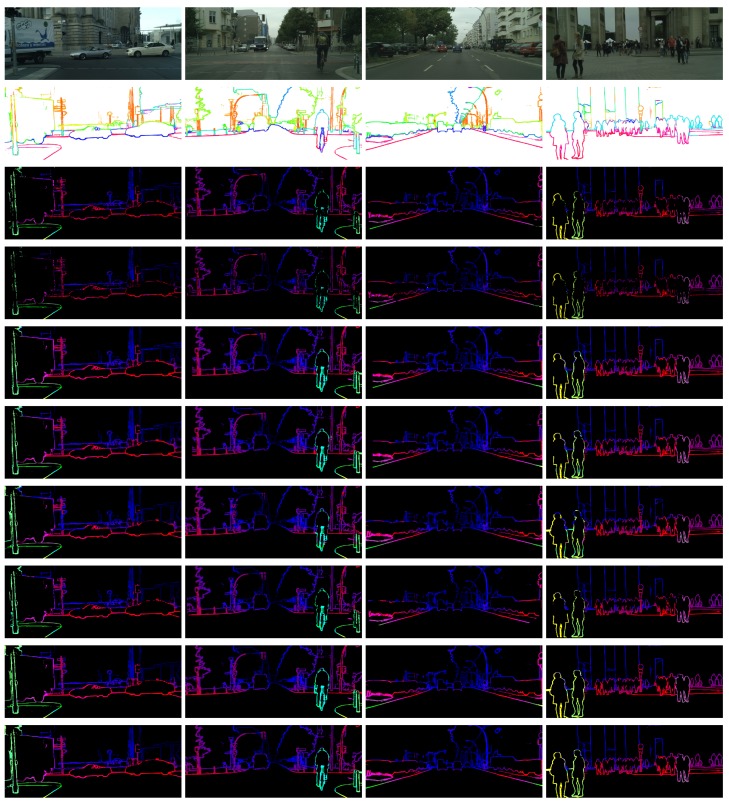
Examples of comparison between our method and other methods. From top to bottom: the left images, the semantic edges, the disparity maps of the following methods separately: SAD + 0.1Census + ADP (LRC), SAD + 0.1Census + ADP (SLRC), SAD + SGM + Refinement, Census + SGM + Refinement, MeshStereo [35], ELAS [36], SPSStereo [37], MC-CNN-fast [5].

The first one is to compare with other dense matching methods, like [16] did. We extract edge parts from the whole disparity maps and only evaluate them. The extracted disparities are denser than our results and can be compared with ours in both qualitative and quantitative evaluation. We contrast our method with SGM [3] using the codes provided in [5]. Different costs are used, and there are two options for SGM: with refinement or without. The refinement strategy also refers interpolation steps in [5]. We also compare our method with MeshStereo [35], ELAS (Efficient LArge-scale Stereo) [36], SPSStereo [37] and MC-CNN-fast [5] with the codes provided by their authors. Results are shown in Table 2 and Figure 8. The best results of our method are superior to others’. Although the running time of our method is at a disadvantage, it can be improved with code optimization and parallel computation.

**Figure 9:**
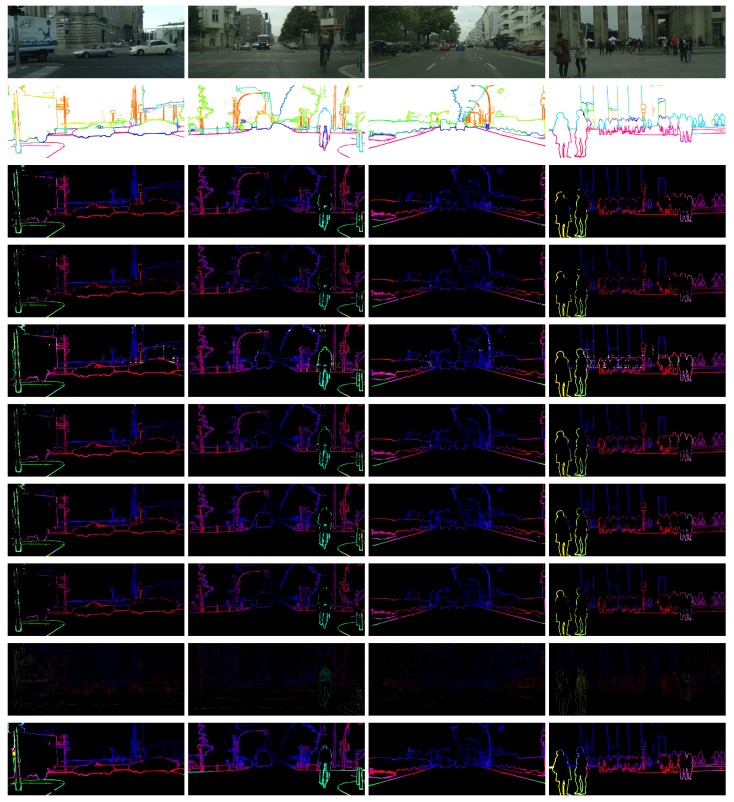
Examples of comparison between our method and SED [19]. From top to bottom: the left images, the semantic edges, the disparity maps of the following methods separately: SAD + ADP (LRC), SAD + ADP (SLRC), Census + ADP (LRC), Census + ADP (SLRC), SAD + 0.1Census + ADP (LRC), SAD + 0.1Census + ADP (SLRC), SED [19], SED after interpolation.

The other one is to compare with SED (Simultaneous Edge Drawing) [19], another sparse matching method based on edges. The Density of SED is 2.78% only. The intersection of SED’s results and our ground truth is very small, so we can not make quantitative evaluation directly. Inspired by the evaluation method of the KITTI stereo dataset [25,26], the initial disparity maps of SED are interpolated according to the interpolation method provided by KITTI. The interpolated dense disparity maps are then evaluated according to the comparison method mentioned above. The results of contrast are shown in Table 3. The qualitative results are shown in Figure 9 for reference, and you can see many obvious mistakes before interpolation. The contours of our method are more clear, and the errors are sporadic.

## 5. Conclusions

In this paper, we present a semantic edge based stereo matching method using an improved adaptive dynamic programming algorithm. The robustness of our method benefits from the reduction of empirical parameters. The single SAD or Census cost can have a considerable effect with our ADP algorithm. When the two costs are combined, we reduce the number of parameters that need to be determined to one. The combined cost can obtain the best results according to our experiments. In ADP, the empirical parameter that is difficult to select in a traditional algorithm is replaced by a range of parameters. The bounds of the range are decided based on the characteristics of the cost by ADP automatically, and the optimal value in the range is obtained based on the disparity or semantic consistency constraint. Different from many other methods, we do not need to set the maximum disparity to improve our performance. The maximum disparity is difficult to estimate in practical applications. Experiments demonstrate that our method offers high accuracy performance and can be put down as one of the top edge-based methods. Furthermore, this study can be extended by considering the constraints between adjacent scan-lines. In our future work, we would like to improve the accuracy of dense stereo matching method with semantic segmentations.

## Figures and Tables

**Table 1:** Comparison between our method and the traditional dynamic programming algorithm.

Algorithm	*Density*	$\mathrm{Error}(\delta_{d}=3)$	$\mathrm{Error}(\delta_{d}=2)$	$\mathrm{Error}(\delta_{d}=1)$	Average Time (s)
SAD + DP ($C_{\mathrm{gap}}=0.0005$)	2.29%	3.29%	**6.26%**	**19.18%**	27.89 (Matlab)
SAD + DP ($C_{\mathrm{gap}}=0.1$)	**10.14%**	14.86%	22.18%	41.35%	**23.62** (Matlab)
SAD + DP ($C_{\mathrm{gap}}=0.9$)	9.39%	37.61%	47.18%	64.25%	24.52 (Matlab)
**SAD + ADP (LRC)**	7.53%	3.35%	6.57%	20.12%	47.15 (Matlab)
**SAD + ADP (SLRC)**	6.01%	**3.26%**	6.47%	20.06%	30.40 (Matlab)
**SAD + ADP (LRC + SLRC)**	6.01%	**3.26%**	6.47%	20.06%	47.98 (Matlab)

**Table 2:** Comparison between our method and other methods.

Algorithm	*Density*	$\mathrm{Error}(\delta_{d}=3)$	$\mathrm{Error}(\delta_{d}=2)$	$\mathrm{Error}(\delta_{d}=1)$	Average Time (s)
SAD + SGM	11.24%	2.90%	5.75%	17.87%	20.42 (GPU)
SAD + SGM + Refinement	11.25%	3.02%	5.89%	18.06%	22.65 (GPU)
Census + SGM	11.23%	3.81%	7.77%	22.85%	20.42 (GPU)
Census + SGM + Refinement	11.24%	3.56%	7.16%	21.97%	22.65 (GPU)
MeshStereo [35]	11.69%	6.88%	11.02%	26.35%	19.81 (C++)
ELAS [36]	10.35%	3.22%	7.42%	29.46%	**0.97** (C++)
SPSStereo [37]	**11.72%**	3.17%	7.44%	28.04%	9.11 (C++)
MC-CNN-fast [5]	**11.72%**	2.79%	6.11%	22.05%	11.61 (GPU)
**SAD + ADP (LRC)**	7.53%	3.35%	6.57%	20.12%	47.15 (Matlab)
**SAD + ADP (SLRC)**	6.01%	3.26%	6.47%	20.06%	30.40 (Matlab)
**Census + ADP (LRC)**	10.33%	12.60%	17.85%	34.22%	1198 (Matlab)
**Census + ADP (SLRC)**	5.85%	5.47%	8.89%	20.82%	831.2 (Matlab)
**SAD + 0.1Census + ADP (LRC)**	8.37%	2.88%	5.58%	17.73%	127.9 (Matlab)
**SAD + 0.1Census + ADP (SLRC)**	6.61%	**2.75%**	**5.44%**	**17.59%**	47.05 (Matlab)

**Table 3:** Comparison between our method and SED [19].

Algorithm	*Density*	$\mathrm{Error}(\delta_{d}=3)$	$\mathrm{Error}(\delta_{d}=2)$	$\mathrm{Error}(\delta_{d}=1)$	Average Time (s)
SED [19]	2.78%	-	-	-	**2.01 (C++)**
SED after interpolation	11.67%	13.86%	20.96%	39.16%	-
**SAD + 0.1Census + ADP (LRC)**	8.37%	2.88%	5.58%	17.73%	127.9 (Matlab)
**SAD + 0.1Census + ADP (SLRC)**	6.61%	**2.75%**	**5.44%**	**17.59%**	47.05 (Matlab)

## Abbreviations

The following abbreviations are used in this manuscript:

AD	Absolute Difference
ADP	Adaptive Dynamic Programming
AR	Augmented Reality
BRISK	Binary Robust Invariant Scalable Keypoints
BP	Belief Propagation
Census	Census Transform
DP	Dynamic Programming
ED	Edge Drawing
ELAS	Efficient LArge-scale Stereo
FAST	Features from Accelerated Segment Test
GC	Graph Cuts
GMS	Grid-Based Motion Statistics
HFL	High-for-Low approach
LRC	Left-Right Consistency
NCC	Normalization Cross Correlation
SAD	the Sum of the Absolute Difference
SED	Simultaneous Edge Drawing
SEM	Semantic Edge Matching
SFM	Structure From Motion
SGM	Semi-Global Matching
SLRC	Semantic Left-Right Consistency
SURF	Speed Up Robust Feature
WTA	Winner-Take-All
